# Supplementary material for: One-pot three component synthesis of substituted dihydropyrimidinones using fruit juices as biocatalyst and their biological studies
Source: PLoS One. 2020 Sep 15;15(9):e0238092. doi: 10.1371/journal.pone.0238092 (PMC7491738; doi:10.1371/journal.pone.0238092)
Supplement: S2 Fig — (DOCX) [file pone.0238092.s002.docx]

**S2 Fig. ^1^HNMR spectrum of compound (4c) (Expanded form 1)**
