# Supplementary figures and images for: One-pot three component synthesis of substituted dihydropyrimidinones using fruit juices as biocatalyst and their biological studies
Source: PLoS One. 2020 Sep 15;15(9):e0238092. doi: 10.1371/journal.pone.0238092 (PMC7491738; doi:10.1371/journal.pone.0238092)

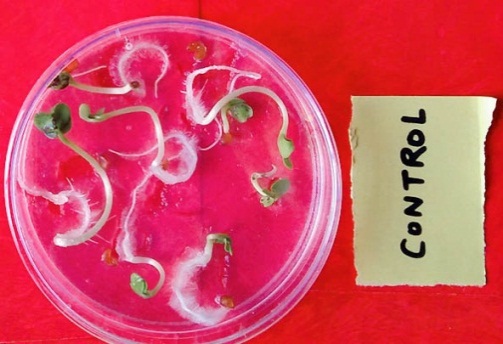


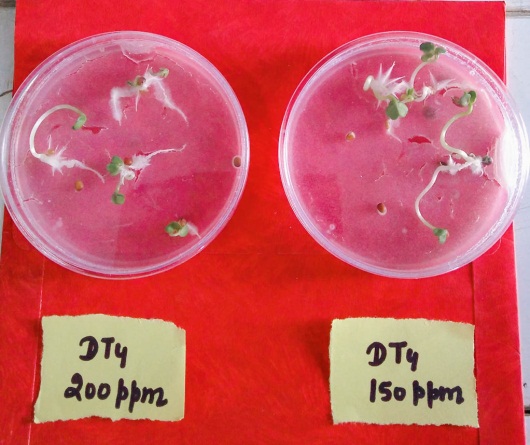

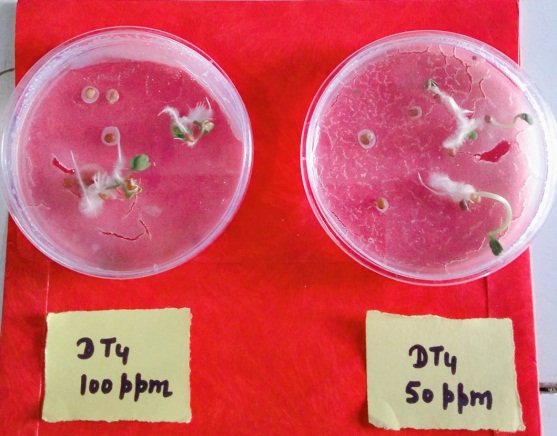


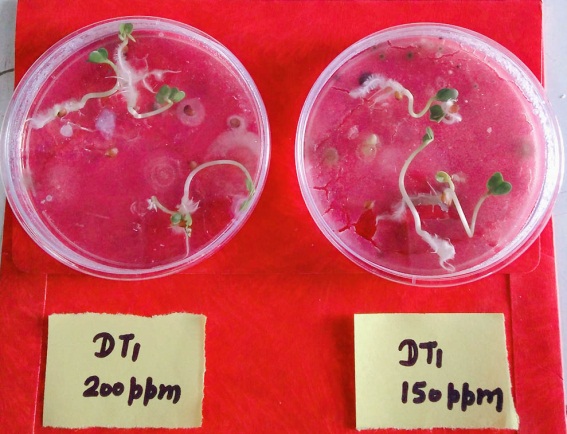

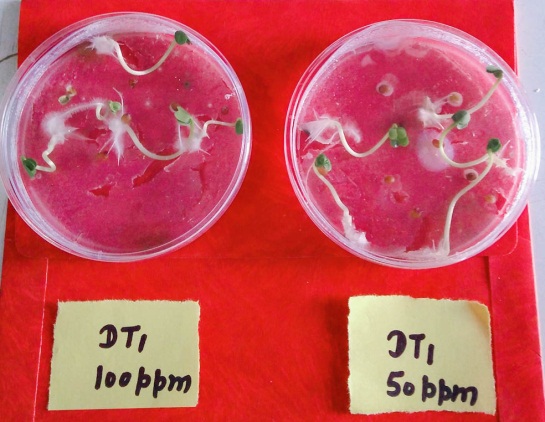


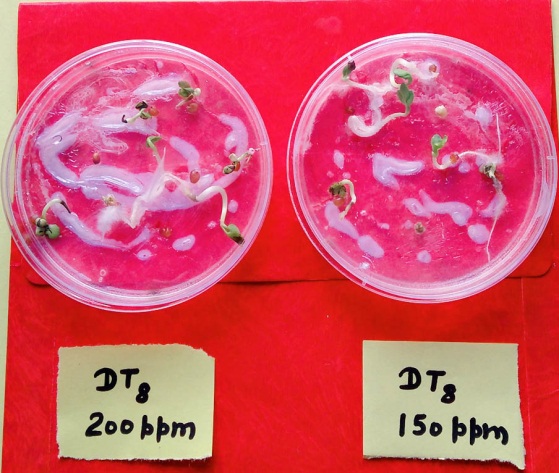

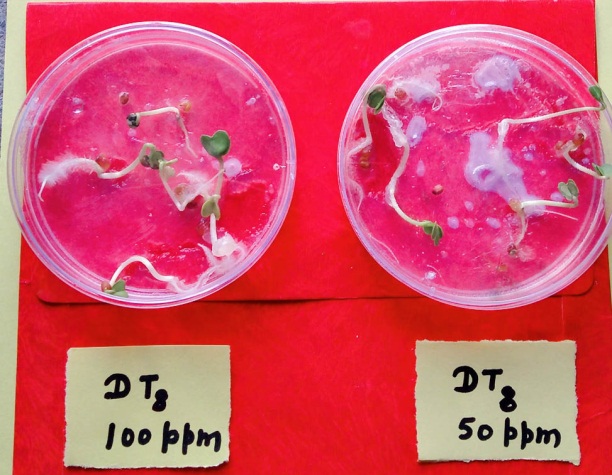


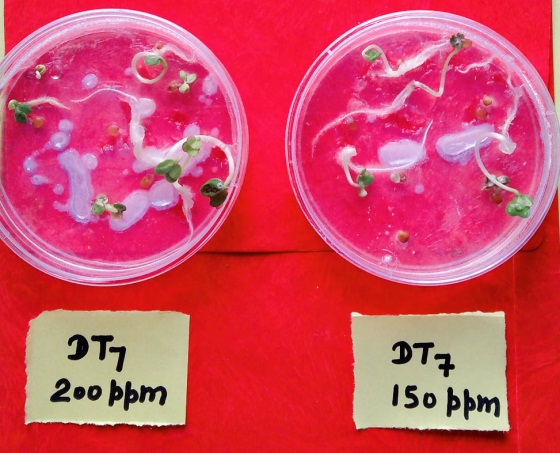

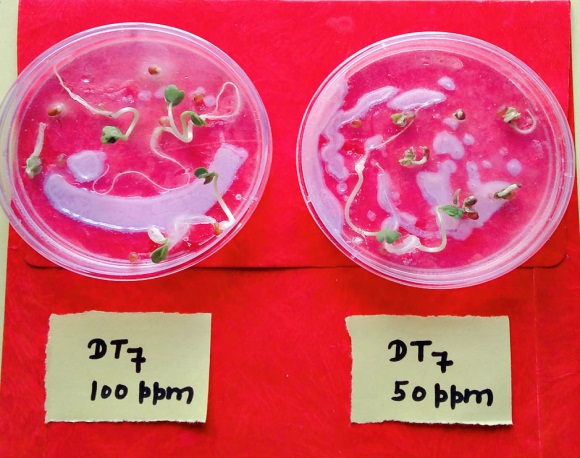


**S11 Fig.** **Herbicidal activity of substituted dihydropyrimidinones (4a-4h)**

Supplement: S11 Fig — (DOCX) [file pone.0238092.s011.docx]

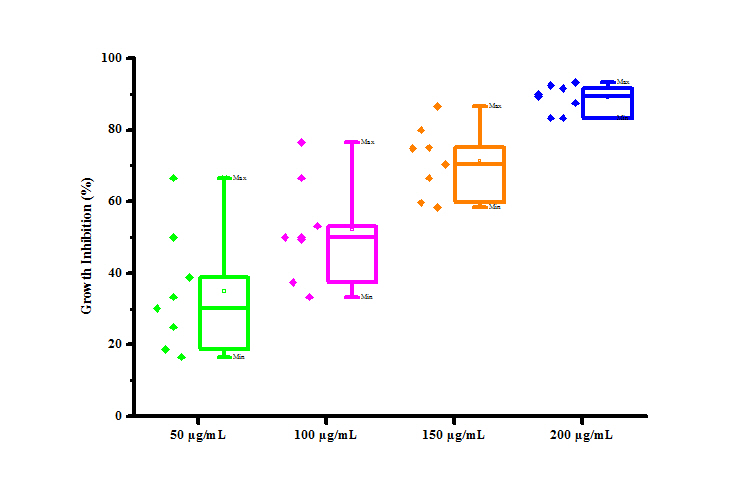
 **S12 Fig. Box plot of substituted dihyropyrimidinones (4a-4h) against *Raphanus sativus* L*.* (root)**

Supplement: S12 Fig — (DOCX) [file pone.0238092.s012.docx]

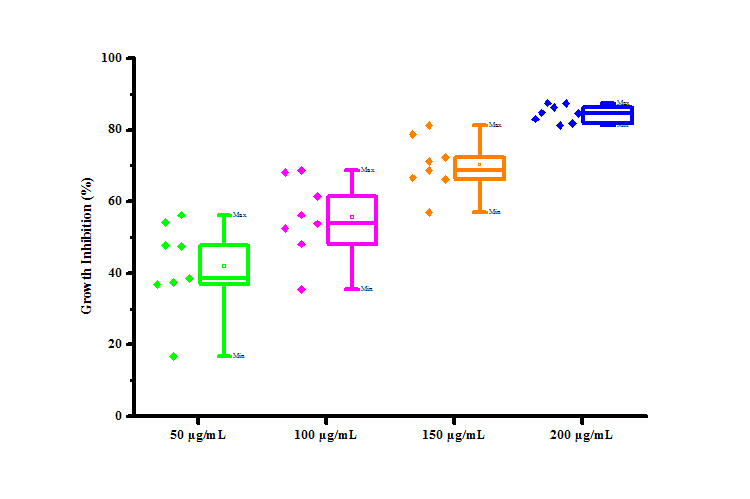
 **S14 Fig. Box plot of substituted dihyropyrimidinones (4a-4h) against *Raphanus sativus* L*.* (shoot)**

Supplement: S14 Fig — (DOCX) [file pone.0238092.s014.docx]

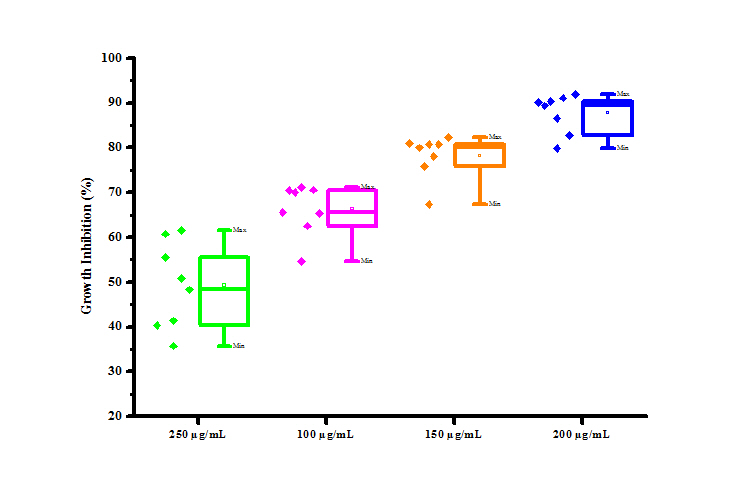
 **S16 Fig. Box plot of substituted dihyropyrimidinones (4a-4h) against *Rhizoctonia solani***

Supplement: S16 Fig — (DOCX) [file pone.0238092.s016.docx]

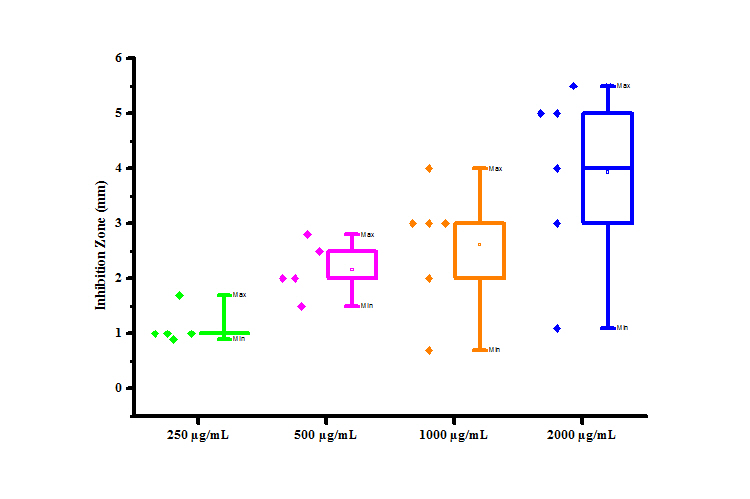
 **S20 Fig. Box plot of substituted dihyropyrimidinones (4a-4h) against *Erwina cartovora***

Supplement: S20 Fig — (DOCX) [file pone.0238092.s020.docx]

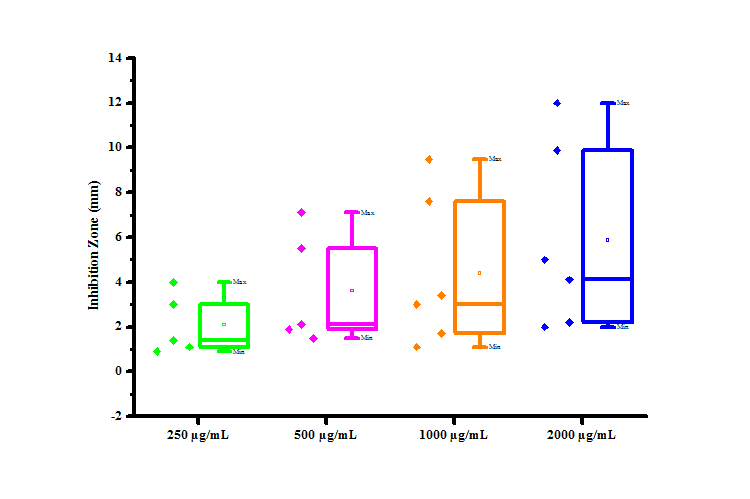
 **S22 Fig. Box plot of substituted dihyropyrimidinones (4a-4h) against *Xanthomonas citri***

Supplement: S22 Fig — (DOCX) [file pone.0238092.s022.docx]

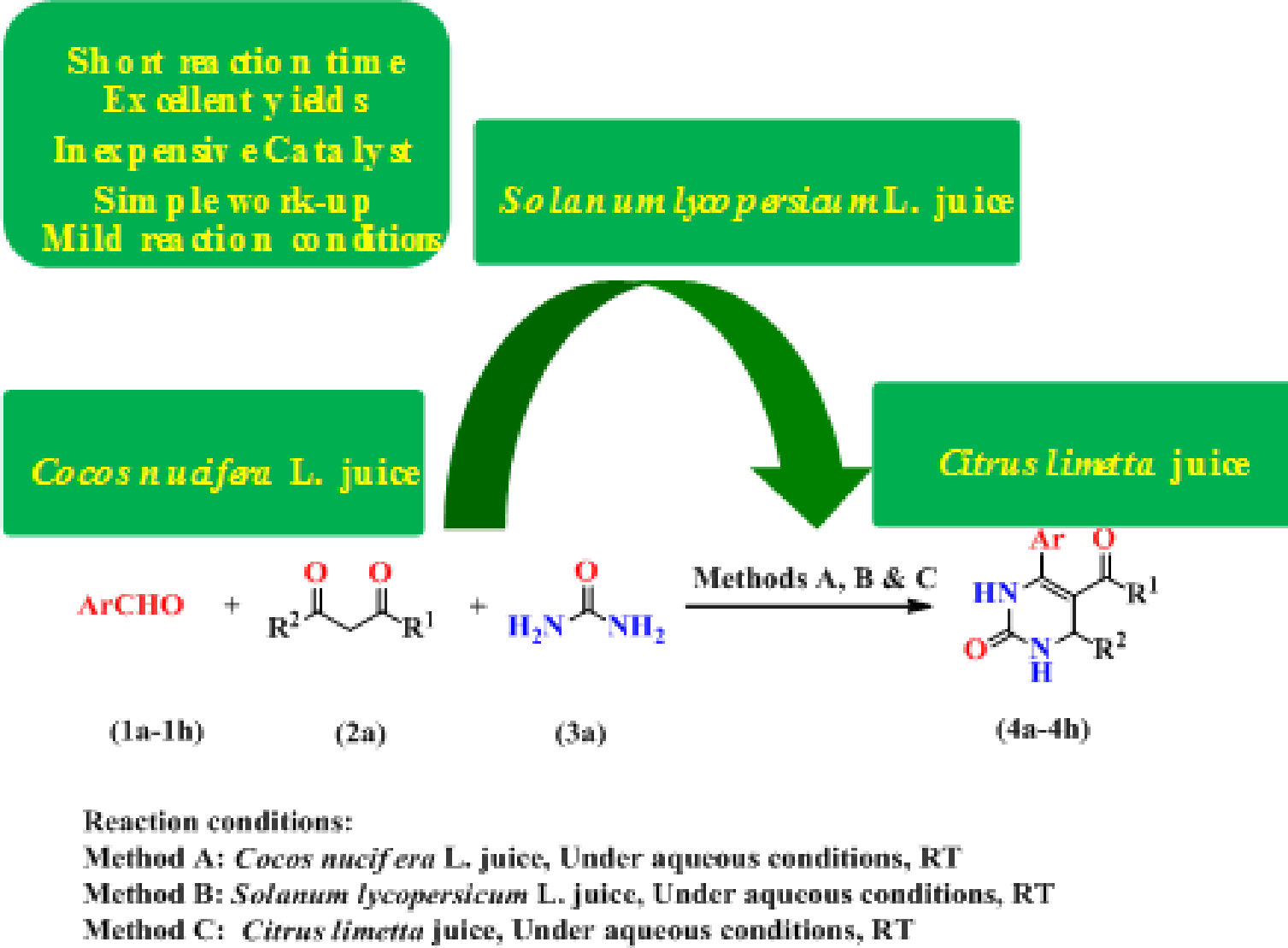

Supplement: Graphical abstract — (TIF) [file pone.0238092.s027.tif]
