# Supplementary material for: One-pot three component synthesis of substituted dihydropyrimidinones using fruit juices as biocatalyst and their biological studies
Source: PLoS One. 2020 Sep 15;15(9):e0238092. doi: 10.1371/journal.pone.0238092 (PMC7491738; doi:10.1371/journal.pone.0238092)
Supplement: S18 Fig — (DOCX) [file pone.0238092.s018.docx]

**
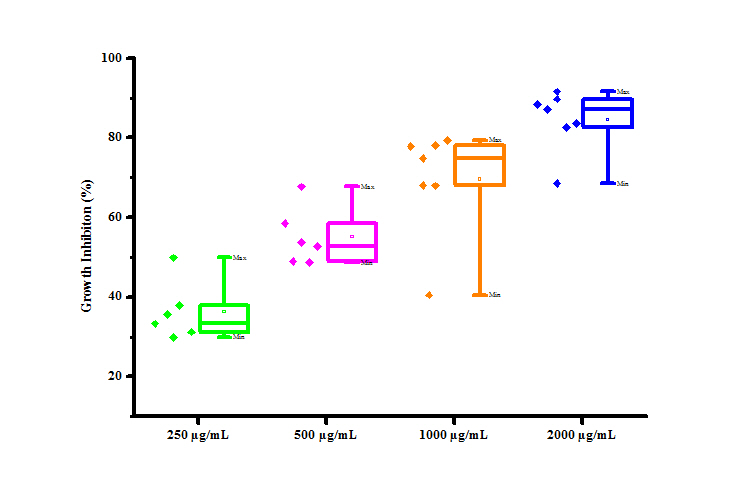
 S18 Fig. Box plot of substituted dihyropyrimidinones (4a-4h) against *Colletotrichum gloeosporioides***
