# Supplementary material for: One-pot three component synthesis of substituted dihydropyrimidinones using fruit juices as biocatalyst and their biological studies
Source: PLoS One. 2020 Sep 15;15(9):e0238092. doi: 10.1371/journal.pone.0238092 (PMC7491738; doi:10.1371/journal.pone.0238092)
Supplement: S1 Table — (DOCX) [file pone.0238092.s024.docx]

**S1 Table. Herbicidal activity of substituted dihydropyrimidinones (4a-4h)**

| **Compounds** | **Growth Inhibition (%)** | | | | | | | |
| --- | --- | --- | --- | --- | --- | --- | --- | --- |
|  | **Root** | | | | **Shoot** | | | |
|  | **50 (µg/mL)** | **100 (µg/mL)** | **150 (µg/mL)** | **200 (µg/mL)** | **50 (µg/mL)** | **100 (µg/mL)** | **150 (µg/mL)** | **200 (µg/mL)** |
| **4a** | 25.00 ± 0.86 | 50.00 ± 0.83 | 66.60 ± 1.05 | 83.33 ± 1.26 | 47.50 ± 0.46 | 56.25 ± 0.85 | 71.25 ± 1.00 | 81.25 ± 1.07 |
| **4b** | 33.33 ± 1.00 | 50.00 ± 2.00 | 75.00 ± 0.99 | 91.66 ± 1.07 | 56.25 ± 0.72 | 68.75 ± 0.74 | 81.20 ± 1.01 | 86.25 ± 0.56 |
| **4c** | 30.20 ± 1.02 | 49.36 ± 1.09 | 74.89 ± 1.24 | 92.36 ± 0.99 | 54.27 ± 0.90 | 68.12 ± 1.15 | 78.78 ± 0.58 | 87.36 ± 0.93 |
| **4d** | 16.60 ± 1.00 | 33.33 ± 0.94 | 58.33 ± 1.34 | 83.33 ± 0.07 | 37.50 ± 0.99 | 52.50 ± 1.21 | 68.75 ± 0.74 | 87.50 ± 1.07 |
| **4e** | 18.67 ± 0.52 | 37.38 ± 0.80 | 59.64 ± 1.27 | 87.52 ± 0.53 | 16.66 ± 1.80 | 35.48 ± 0.68 | 56.97 ± 0.76 | 81.87 ± 1.08 |
| **4f** | 38.72 ± 0.90 | 53.16 ± 0.99 | 70.48 ± 1.00 | 89.38 ± 0.93 | 36.78 ± 0.67 | 48.18 ± 0.85 | 66.66 ± 0.74 | 84.78 ± 1.15 |
| **4g** | 66.60 ± 0.53 | 76.60 ± 1.00 | 86.60 ± 0.47 | 93.33 ± 1.46 | 47.69 ± 0.78 | 61.50 ± 0.85 | 72.30 ± 1.07 | 84.61 ± 1.06 |
| **4h** | 50.00 ± 0.82 | 66.60 ± 1.12 | 80.00 ± 0.84 | 90.00 ± 1.26 | 38.46 ± 1.49 | 53.80 ± 1.00 | 66.15 ± 0.86 | 83.07 ± 1.71 |

**All values are mean ± S.D.**
