# Supplementary material for: One-pot three component synthesis of substituted dihydropyrimidinones using fruit juices as biocatalyst and their biological studies
Source: PLoS One. 2020 Sep 15;15(9):e0238092. doi: 10.1371/journal.pone.0238092 (PMC7491738; doi:10.1371/journal.pone.0238092)
Supplement: S2 Table — (DOCX) [file pone.0238092.s025.docx]

**S2 Table. Antifungal activity of substituted dihydropyrimidinones (4a-4h)**

| **Compounds** | **Growth inhibition (%)** | | | | | | | |
| --- | --- | --- | --- | --- | --- | --- | --- | --- |
|  | **Fungi** | | | | | | | |
|  | ***Rhizoctonia solani* (conc.) µg/mL** | | | | ***Colletotrichum gloeosporioides* (conc.) µg/mL** | | | |
|  | **250** | **500** | **1000** | **2000** | **250** | **500** | **1000** | **2000** |
| **4a** | 61.53 ± 0.90 | 71.15 ± 1.03 | 80.76 ± 2.01 | 86.53 ± 1.08 | 37.89 ± 1.16 | 53.80 ± 0.77 | 78.18 ± 0.16 | 89.78 ± 1.09 |
| **4b** | 41.35 ± 0.89 | 62.56 ± 0.72 | 78.13 ± 0.70 | 89.38 ± 1.04 | 35.63 ± 1.19 | 58.45 ± 1.16 | 74.89 ± 1.10 | 87.12 ± 0.96 |
| **4c** | 50.87 ± 1.04 | 69.99 ± 0.78 | 80.00 ± 2.67 | 91.13 ± 1.95 | 49.90 ± 1.05 | 67.70 ± 0.50 | 79.45 ± 1.10 | 91.58 ± 1.06 |
| **4d** | 40.38 ± 1.53 | 65.38 ± 1.02 | 80.76 ± 1.76 | 90.38 ± 1.42 | 31.25 ± 1.00 | 48.70 ± 1.26 | 67.93 ± 0.98 | 82.56 ± 0.61 |
| **4e** | 35.70 ± 1.00 | 54.68 ± 0.37 | 67.30 ± 1.61 | 79.89 ± 2.26 | a | a | a | a |
| **4f** | 55.50 ± 1.64 | 70.50 ± 2.45 | 80.98 ± 2.26 | 92.00 ± 1.02 | 33.30 ± 1.59 | 52.78 ± 1.39 | 77.80 ± 0.99 | 88.45 ± 0.61 |
| **4g** | 48.27 ± 1.92 | 65.51 ± 1.62 | 75.86 ± 2.41 | 82.75 ± 1.37 | 29.96 ± 1.36 | 49.00 ± 0.71 | 68.12 ± 0.92 | 83.59 ± 0.45 |
| **4h** | 60.78 ± 1.84 | 70.58 ± 1.61 | 82.35 ± 1.06 | 90.19 ± 0.64 | a | a | 40.50 ± 1.70 | 68.54 ± 0.91 |

**All values are mean ± S.D. a: No Growth inhibition**
