# Supplementary material for: One-pot three component synthesis of substituted dihydropyrimidinones using fruit juices as biocatalyst and their biological studies
Source: PLoS One. 2020 Sep 15;15(9):e0238092. doi: 10.1371/journal.pone.0238092 (PMC7491738; doi:10.1371/journal.pone.0238092)
Supplement: S3 Table — (DOCX) [file pone.0238092.s026.docx]

**S3 Table. Antibacterial activity of substituted dihydropyrimidinones (4a-4h)**

| **Compounds** | **Inhibition Zone (mm)** | | | | | | | |
| --- | --- | --- | --- | --- | --- | --- | --- | --- |
|  | **Bacteria** | | | | | | | |
|  | ***Erwinia cartovora*** **(conc.) µg/mL** | | | | ***Xanthomonas citri*** **(conc.) µg/mL** | | | |
|  | **250** | **500** | **1000** | **2000** | **250** | **500** | **1000** | **2000** |
| **4a** | a | a | a | a | a | a | a | a |
| **4b** | 0.90 ± 0.07 | 1.50 ± 0.10 | 2.00 ± 0.30 | 3.00 ± 0.40 | 1.40 ± 0.20 | 2.10 ± 0.18 | 3.40 ± 0.20 | 4.10 ± 0.45 |
| **4c** | 1.70 ± 0.30 | 2.80 ± 0.35 | 4.00 ± 0.41 | 5.00 ± 0.45 | 0.90 ± 0.07 | 1.50 ± 0.28 | 1.70 ± 0.15 | 2.20 ± 0.47 |
| **4d** | a | a | a | a | a | a | a | a |
| **4e** | a | a | 0.70 ± 0.07 | 1.10 ± 0.26 | 3.00 ± 0.47 | 5.50 ± 0.30 | 7.60 ± 0.30 | 9.90 ± 0.43 |
| **4f** | 1.00 ± 0.18 | 2.00 ± 0.45 | 3.00 ± 0.15 | 5.00 ± 0.55 | 4.00 ± 0.50 | 7.10 ± 0.36 | 9.50 ± 0.26 | 12.0 ± 0.40 |
| **4g** | 1.00 ± 0.09 | 2.50 ± 0.35 | 3.00 ± 0.16 | 5.50 ± 0.50 | a | a | 1.10 ± 0.20 | 2.00 ± 0.30 |
| **4h** | 1.00 ± 0.12 | 2.00 ± 0.17 | 3.00 ± 0.15 | 4.00 ± 0.45 | 1.10 ± 0.22 | 1.90 ± 0.40 | 3.00 ± 0.26 | 5.00 ± 0.50 |

**All values are mean ± S.D.**
