# Supplementary material for: One-pot three component synthesis of substituted dihydropyrimidinones using fruit juices as biocatalyst and their biological studies
Source: PLoS One. 2020 Sep 15;15(9):e0238092. doi: 10.1371/journal.pone.0238092 (PMC7491738; doi:10.1371/journal.pone.0238092)
Supplement: Attachment — Submitted filename: Response file.docx [file pone.0238092.s029.docx]

**R/Sir all the suggestions according to reviewer comments incorporated successfully in revised (R2** **[PONE-D-20-15280R1]) manuscript. Thanks to editorial staff and reviewer for their timely support and necessary suggestions for making revised manuscript more fruitful. All suggestions were shown in manuscript by track change mode. Respond to reviewer were shown in Blue colour.**

**Comments from the Editors and Reviewers:**

Comments to the Author

Simplify the abstract

**Answer to Reviewer:**

- **Authors simplify the abstract according to reviewer suggestions in revised manuscript.**

Comments to the Author

Cite appropriate scientific literature including the examples recommended by the reviewers

**Answer to Reviewer:**

- **Authors cited the appropriate scientific literature in the manuscript. For e.g.**

1. **Patil S, Jadhav SD, Deshmukh MB. Natural acid catalyzed multi-component reactions as a green approach. Arch. Apll. Sci. Res., 2011 3(1):203-208.**
2. **Tamuli KJ, Dutta D, Nath S, Bordoloi M. A Greener and Facile Synthesis of Imidazole and Dihydropyrimidine Derivatives under Solvent‐Free Condition Using Nature‐Derived Catalyst. Chemistry Select. 2017 2(26):7787-7791.**
3. **Fonseca AM, Monte FJ, Maria da Conceição F, de Mattos MC, Cordell GA, Braz-Filho R, Lemos TL. Coconut water (Cocos nucifera L.)—A new biocatalyst system for organic synthesis. J. Mol. Catal. B Enzym. 2009 57(1-4):78-82.**
4. **Nazeruddin GM, Shaikh YI. Tamarind juice catalyzed one pot synthesis of dihydropyrimidinone and thione under ultrasound irradiation at ambient conditions: A green approach. Der Pharmacia Sinica., 2014 5(6):64-68.**
5. **Ramu E, Kotra V, Bansal N, Varala R, Adapa SR. Green approach for the efficient synthesis of Biginelli compounds promoted by citric acid under solvent-free conditions. Rasayan Journal of Chem., 2008 1(1):188-194.**

Comments to the Author

Correct solvent free to ‘under aqueous conditions’

**Answer to Reviewer:**

- **Authors replaced solvent-free to ‘under aqueous conditions’ in revised manuscript.**

Comments to the Author

Carry out quantitative measurement of natural acids and proteins as requested by reviewer 1

**Answer to Reviewer:**

- **Authors in their experiment not quantify natural acids and proteins.**

Comments to the Author

Discuss the nature of the catalyst- ascorbic acid, other acids, metal ions etc.

**Answer to Reviewer:**

- **Authors used nature derived catalyst *viz*. *Cocos nucifera* L. juice, *Solanum lycopersicum* L. juice and *Citrus limetta* juice in their study. All these catalyst contain various organic acids mainly ascorbic acid, citric acid and malic acid etc. All these acids are weak organic acid. Ascorbic acid is an organic compounds called hexuronic acid and it dissolves well in water to give mildly acidic solutions. It is mild reducing agent.**

Comments to the Author

Follow the literature precedents for discussion on the mechanism

**Answer to Reviewer:**

- **Authors cited the literature for discussion on the mechanism in revised manuscript.**

Comments to the Author

Write clear experimental procedures to enable reproducibility by others

**Answer to Reviewer:**

- **Authors improved the experimental procedure in revised manuscript. Now the experimental procedure becomes clearer.**

Comments to the Author

Provide detailed characterization of compounds including copies of NMR spectra to confirm compound purity

**Answer to Reviewer:**

- **Authors provided detailed characterization (^I^HNMR and FTIR) of some selected compounds to confirm compound purity.**
- **Compounds name and structure also provided in NMR and FTIR chart file.**
- **Authors revised the manuscript according to PLOS ONE style.**
- **Authors deleted the funding information in the Acknowledgments section in revised manuscript.**
- **Authors revised the graphical abstract according to reviewer suggestions.**
- **Authors successfully added the details of colleague who edited the manuscript in revised manuscript.**
- **Authors successfully added the financial disclosure statement “Authors receive no specific funding” in revised manuscript.**
- **Authors successfully deleted the copy right images from the graphical abstract and revised the graphical abstract according to reviewer suggestions in revised manuscript.**
- **Authors successfully added Data availability statement in revised manuscript.**
- **Authors successfully uploaded** **minimal underlying data set as either Supporting Information files in revised manuscript.**
- **Authors thoroughly copyedit the manuscript for language usage, spelling, and grammar.**
- **In manuscript all the graph made by OriginPro 8 software and standard deviation was also included in graphical representation to make manuscript more scientifically.**
- **All the structure in manuscript made by using ChemDraw Ultra 12.0 software.**
- **Manuscript is now accordance to PLOS ONE style.**
- **Thanks to the editorial board and reviewers for necessary suggestions regarding the manuscript.**

**Answer to Reviewer Regarding [PONE-D-20-15280R1]:**

- **In revised manuscript author had cited relevant papers that explain the utility of fruit juices in organic synthesis in introduction part of manuscript and detail of references are given below:**
- **Pramanik T, Pathan AH, Gupta R, Singh J, Singh S. Exploring the utility of fruit juices as green medium for Biginelli reaction. Res. J. Pharm. Bio. Chem. Sci. 2013 5(5):444-449.**
- **Pal R. Fruit juice; A natural, green and biocatalyst system in organic synthesis. Open J. Org. Chem. 2013 1(4);47-56.**
- **Authors also deleted the poor quality references from the manuscript. Quality reference which explain the mechanism of reaction was include in revised manuscript and details reference given as below:**
  - **Sharma N, Sharma UK., Kumar R, Richa, Sinha AK. Green and recyclable glycine nitrate (GlyNO3) ionic liquid triggered multicomponent Biginelli reaction for the efficient synthesis of dihydropyrimidinones. RSC Adv. 2012, 28;1-4.**
- **Authors feel that revised manuscript acceptable for publication and authors declared that there is no conflict of interest regarding publication of this paper.**
- **The revised manuscript is technically sound, scientifically written. Experiments related to biological activity were performed in triplicate and conclusion is well written based on data presented in manuscript.**
- **Statistical analysis (Standard deviation) mentioned in table and graph for making revised manuscript more informative.**
- **All relevant data are within the paper and its supporting information files. Authors made all data underlying the findings in their manuscript fully available.**
- **Authors presented the manuscript in intelligible fashion and written in standard English language.**
- **Financial disclosure statement was also given in cover letter.** **Authors received no specific funding for this study. The funder has no role in study design, data collection and analysis, decision to publish or preparation of the manuscript.**
- **All the figures in revised manuscript meet PLOS requirements, according to Preflight Analysis and Conversion Engine (PACE).**
- **Revised manuscript fully meets PLOS ONE’s publication criteria.**
- **Thanks to the editorial board and reviewers for necessary suggestions regarding the manuscript. Please consider all the points mentioned above and if reviewer required any more revision then inform by email.**
